# Antibacterial Hydrogels

**DOI:** 10.1002/advs.201700527

**Published:** 2018-02-22

**Authors:** Shuqiang Li, Shujun Dong, Weiguo Xu, Shicheng Tu, Lesan Yan, Changwen Zhao, Jianxun Ding, Xuesi Chen

**Affiliations:** ^1^ Department of Bone and Joint Surgery The First Hospital of Jilin University Changchun 130022 P. R. China; ^2^ Key Laboratory of Polymer Ecomaterials Changchun Institute of Applied Chemistry Chinese Academy of Sciences Changchun 130022 P. R. China; ^3^ VIP Integrated Department School and Hospital of Stomatology Jilin University Changchun 130021 P. R. China

**Keywords:** antibacterial properties, biomedical applications, hydrogels

## Abstract

Antibacterial materials are recognized as important biomaterials due to their effective inhibition of bacterial infections. Hydrogels are 3D polymer networks crosslinked by either physical interactions or covalent bonds. Currently, hydrogels with an antibacterial function are a main focus in biomedical research. Many advanced antibacterial hydrogels are developed, each possessing unique qualities, namely high water swellability, high oxygen permeability, improved biocompatibility, ease of loading and releasing drugs, and structural diversity. Here, an overview of the structures, performances, mechanisms of action, loading and release behaviors, and applications of various antibacterial hydrogel formulations is provided. Furthermore, the prospects in biomedical research and clinical applications are predicted.

## 1 Introduction

Since the first discovery of penicillin in 1928,^1^ antibiotics have been widely used in the antibacterial field. With the development of public hygiene and biomedical technology, many infections have been effectively suppressed or even conquered, and the quality of life for human beings has been significantly improved. However, a serious issue that still remains is that the use of antibiotics has led to the emergence of multidrug resistant microorganisms, which are very difficult to combat.^2^ This has led to over 13 million people dying per year from infectious diseases worldwide.^3^ What was the most disappointing was that the corresponding antibiotic‐resistant bacteria emerged almost immediately after the advanced antibiotics were approved, e.g., the fidaxomicin‐resistant *Enterococci* (K‐1476) and the methicillin‐resistant *Staphylococcus aureus* (*S. aureus*)(MRSA).^4^, ^5^, ^6^

**Figure 1:**
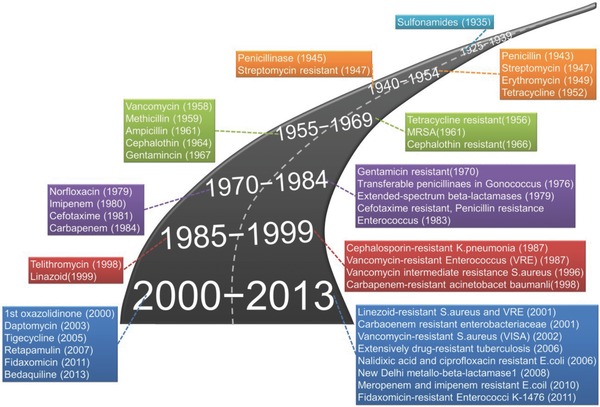
History of antibacterial agents and acquisition of resistance by microorganism.

**Figure** **1** shows the history of the development of antibacterial agents followed by the acquisition of resistance by microorganism. Synthetic antibacterial agents, such as salicylate (SAL), chlorhexidine (CHX), isothiazolinone (ITZ), thiosemicarbazone (TSC), octenidine (OCT), and quaternary ammonium (QA) compounds, also face constant threats because of the drug resistance acquired by microorganisms.^7^ Additionally, conventional antibiotics also face other problems, such as solubility, overdose, and cytotoxicity. Therefore, an efficient and safe drug delivery system, which can reduce the risk of bacterial drug‐resistance and regulate the toxicity of antibacterial drugs, is in high demand.

Challenged by the ever‐growing threats from drug‐resistant pathogenic microorganisms, researchers have been studying various advanced antibacterial materials. Among them, heavy metal ions and natural extracts were discovered and applied in the antibacterial field. However, these materials can inhibit and kill not only the pathogenic microbes, but also normal cells in the human body, which limits the potential applications for these materials.

Hydrogels are a form of 3D porous materials, which consist of polymer chains with either physical or chemical crosslinking.^8^, ^9^, ^10^, ^11^ Hydrogels have been extensively studied as an alternative material for antibacterial applications. By carefully selecting monomers and crosslinkers, the desired abilities of hydrogels, such as the hydrophilicity and porosity, can be developed for antibacterial applications. Moreover, some types of hydrogels also have an inherent antibacterial property.

**Scheme 1:**
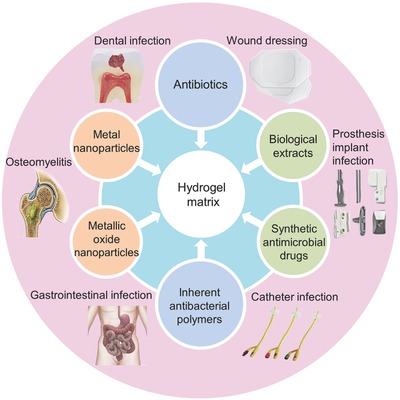
Compositions, performances, and applications of antibacterial hydrogels.

According to the classification of hydrogel matrices and the antibacterial agents, the antibacterial hydrogels are divided into three types: (i) inorganic nanoparticle‐containing hydrogels, (ii) antibacterial agent‐containing hydrogels, and (iii) hydrogels with inherent antibacterial capabilities. This article will describe the syntheses, performances, action mechanisms, loading and release behaviors, and applications of antibacterial hydrogels, as depicted in **Scheme** **1**.

## 2 Inorganic Antibacterial Agent‐Incorporated Hydrogels

**Figure 2:**
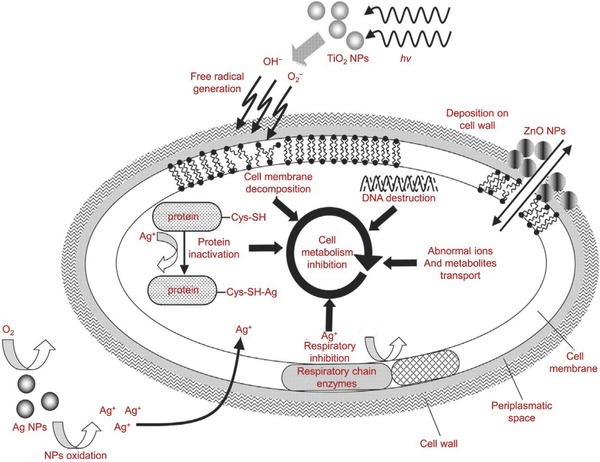
Antibacterial mechanisms of metal and metallic oxide nanoparticles. Reproduced with permission.^12^ Copyright 2013, Elsevier.

Inorganic antibacterial materials mainly include metal ions and metallic oxide nanoparticles. Commonly used metal/metal ions include, but are not limited to, silver (Ag), gold (Au), and copper (Cu). Metallic oxide metal nanoparticles that are utilized include zinc oxide (ZnO), titanium dioxide (TiO_2_), and nickel oxide. Currently, the most widely used inorganic antibacterial materials are silver nanoparticles (Ag NPs) and ZnO NPs. Inorganic antibacterial material‐loaded hydrogels can not only enhance the antibacterial properties, but can also maintain antibacterial activity for a long period of time, which reduces the likelihood of bacterial resistance arising. **Figure** **2** illustrates the possible antibacterial mechanisms of the metal and metallic oxide nanoparticles.^12^ To summarize, the nanoparticles cause damage to bacterial cell membranes or detrimental alterations to organelles. It should be emphasized, however, that some of these mechanisms are speculative and require further discussion and demonstration.

### 2.1 Metal Nanoparticle‐Loaded Hydrogels

#### 2.1.1 Silver Nanoparticle‐Loaded Hydrogels

Since thousands of years ago, even before the word “microorganism” was established, Ag was already regarded as an antibacterial agent. Ag powder was first documented in medical history to be applied in wound healing for the treatment of ulcers by Hippocrates. Ag continues to play an important role in biomedical applications, such as for wound dressings, textiles, and bone implants. With the development of nanoscience and nanotechnology, the recent applications of Ag are mainly in the form of nanoparticles.^13^ Ag NPs are emerging as an efficient antibacterial agent, although the mechanisms remain unclear. The most accepted hypothesis is that the silver ion (Ag^+^) can bind to the bacterial cell membrane through the interaction between Ag^+^ and the thiol group in proteins on the cell membrane, thus affecting the bacterial cell's viability by inhibiting the replication of DNA (Figure 2).

Hydrogels containing Ag NPs include two types of matrices: (i) the natural polymers or modified natural polymers and (ii) synthetic polymers. The polysaccharides play an important role in the natural hydrogel matrix. Alginate is one of the linear natural polysaccharides that can form hydrogels via ionic interactions with Ca^2+^. Stojkovska et al. incorporated Ag NPs into sodium alginate (SA) microbeads through an electrochemical procedure, which efficiently released Ag NPs and/or Ag^+^ and showed antibacterial activity against *S. aureus*.^14^ More specifically, the maximal concentration of released Ag from SA microbeads was about 0.3 × 10^−3^ m, which killed 95.8% of the bacteria after 1 h of coincubation. These results showed that SA was successfully utilized for preparation of SA microbeads incorporated with Ag NPs as antimicrobial agents against *S. aureus*. Madhusudana Rao et al. further contributed to this research by creating SA‐based semi‐interpenetrating polymer network hydrogels for the incorporation of Ag NPs. The research showed that Ag nanocomposite hydrogels could be used for biomedical applications, such as wound dressings and even water purification. Furthermore, Neibert et al. described a method to enhance the mechanical strength of SA hydrogel loaded with Ag NPs. The calcium‐ or *N*,*N*‐methylenebisacrylamide‐crosslinked SA fibers were loaded with Ag NPs, which could be applied to wound dressings or utilized for healing purposes.^15^, ^16^ All the hydrogels loaded with Ag NPs showed good antibacterial activity against Gram‐negative (G−) bacterium *Escherichia coli* (*E. coli*).^17^, ^18^ The natural and biodegradable SA nanocomposite hydrogels showed a sustained release of Ag and a long‐term antibacterial activity.

Chitosan (CS) and chitin (CT) have inherent antibacterial and metal‐binding properties. CS‐ or CT‐based hydrogels like CS/2‐GP/nanosilver hydrogels (GP, glycerophosphate)^19^ and silver molybdate NANOPARTICLEs/CT matrix, are also commonly used for antibacterial applications. Ag_2_Mo_2_O_7_/CT hydrogels provide green synthesis processes and excellent antibacterial abilities against *E. coli*.^20^ With the help of CS or CT, nanosilver hydrogels had enhanced the efficacy and reduced the toxicity. Reddy et al. demonstrated that the natural and nontoxic gelatin contributed to anchoring and stabilizing Ag NPs. Thus, they prepared poly(gelatin acrylamide) silver nanocomposite hydrogels for the inactivation of bacteria.^21^

In addition to utilizing natural polymer for natural polymer‐ or modified natural polymer‐based hydrogels as antibacterial matrices, many synthetic polymers have also been applied to fabricate the Ag NP‐loaded hydrogels, such as polyacrylamide (PAAm), poly(acrylic acid) (PAA), poly(ethylene glycol) (PEG), poly(vinyl alcohol) (PVA), poly(*N*‐vinyl‐2‐pyrrolidone) (PVP), as well as short peptides and their derivatives. The main advantage of using these hydrogels as a matrix is that the morphologies and sizes can be easily controlled by changing the amount of crosslinkers and monomers in the hydrogel network.^22^, ^23^, ^24^ For example, compared with Ag NPs alone and Ag^+^‐bonded hydrogels, the Ag NP‐loaded PAAm/PVA hydrogels fabricated by Varaprasad and co‐workers exhibited a higher antibacterial activity toward *E. coli*. This was because the Ag NPs in the hydrogels had good dispersion capability throughout the hydrogel network. Styrene sulfonic acid sodium salt was incorporated into the hydrogels to form the Ag NP‐loaded hydrogel composed of poly(acrylamide‐*co*‐styrene sulfonic acid sodium salt) and CS, which could combat the most sensitive strains of *Bacillus subtilis* (*B. subtilis*).^23^

**Figure 3:**
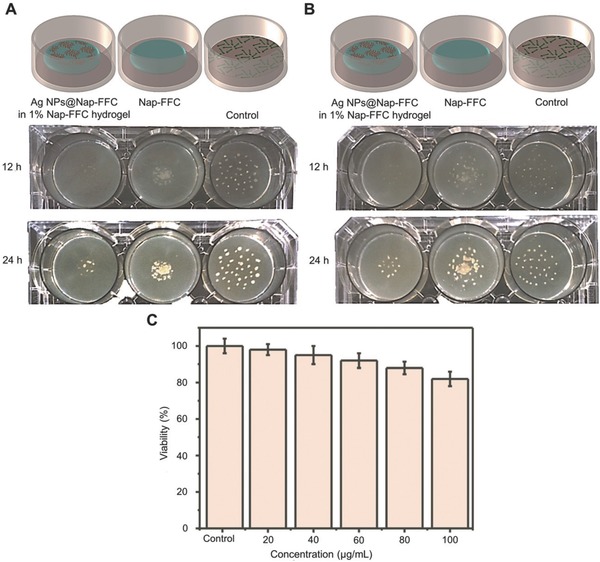
Biocompatible Ag NP‐derived tripeptide supramolecular hydrogel for antibacterial wound dressings. A,B) Schematic illustration of antibacterial tests of Nap‐FFC and Ag NP@Nap‐FFC hydrogels. C) Cytotoxicity assay of Ag NP@Nap‐FFC nanocomposites toward HeLa cells. Reproduced with permission.^28^ Copyright 2016, Royal Society of Chemistry.

In order to increase the stability and dispersity of metal nanoparticles in aqueous media and control the nanostructure, semi‐interpenetrating network hydrogels composed of Pluronic and PAAm were simultaneously prepared by free radical crosslinking polymerization and served as nanoreactors for the synthesis of Ag NPs.^22^ The Ag NP‐loaded hydrogels formed by mixing of PAAm with itaconic acid (IA)^25^ or starch (ST)^26^ were also reported to possess good antibacterial properties while providing a green synthesis process. Boonkaew et al. synthesized 2‐acrylamido‐2‐methylpropane sulfonic acid sodium salt hydrogels containing Ag NPs. The hydrogel with 5.0 × 10^−3^ m Ag NPs displayed the highest antimicrobial activity for wound infection prevention without cytotoxicity.^27^ Simon et al. synthesized a *N*‐terminally 2‐(naphthalen‐6‐yl)acetic acid‐protected Phe‐Phe‐Cys peptide (Nap‐FFC) hydrogel, which incorporated Ag NPs and showed inhibition against both Gram‐positive (G+; MRSA) and G− (i.e., *Acinetobacter baumannii*) bacteria.^28^ It is important to note that the hydrogels had excellent biocompatibilities compared to human cervical carcinoma HeLa cells (**Figure** **3**). All these hydrogels demonstrated noticeable antibacterial properties, which gave researchers more confidence on the exploitation of Ag NP hydrogels.

Hydrogel matrices obtained from different synthesis processes possess differing characteristics. P(AA‐*co*‐PEGMA)/Ag NP composite hydrogels were developed by Lee and Tsao, offering a promising bioadhesive patch or wound dressing material (PEGMA, poly(ethylene glycol) methyl ether acrylate).^29^ Ag NP‐coated CS wafer‐loaded PVA hydrogels (PVA/Ag–CHW hydrogels) were formulated by a sonication technique and then used as a wound dressing. The PVA/Ag–CHW hydrogels improved the re‐epithelialization, increased angiogenesis, and enhanced wound healing without any undesirable inflammatory response.^30^ A thermoplastic hydrogel was synthesized from multiblock PEG–POSS (POSS; poly(hedral oligosilsesquioxane)) polyurethanes by Wu et al.^31^ Without Ag, the hydrogel exhibited the most rapid and extensive biofilm formation. Meanwhile, the Ag‐containing nanofibrous hydrogel possessed outstanding biofilm resistance and antibacterial property that lasted over 14 days. PVA/PVP‐based hydrogels fabricated by Eid et al. containing Ag NPs were reported to be uniformly distributed and highly stable.^32^ The pH‐sensitive poly(methyl methacrylate‐*co*‐methacrylic acid)/Ag NP hydrogels synthesized by a free radical crosslinking copolymerization approach have the potential to be utilized as an antibacterial biomaterial.^33^

All these hydrogels mentioned above displayed antibacterial ability against *E. coli*, *S. aureus*, *Pseudomonas aeruginosa* (*P. aeruginosa*), and *B. subtilis*. Additionally, an antibacterial coating made of poly(l‐lysine)/hyaluronic acid multilayer films and liposomes loaded with Ag^+^ was also explored.^34^ The strong antibacterial effect was attributed to the diffusion of Ag^+^ from the AgNO_3_ coating, which resulted in Ag^+^ aggregation around the membranes of bacteria. Moreover, other small antibacterial molecules like antibiotics could be loaded into hydrogels using this method to accomplish the goal of delayed drug delivery. Furthermore, there are mussel‐inspired Ag NP hydrogels synthesized with water‐soluble PEG, which contain reactive catechol moieties inspired by mussel adhesive proteins. Mussels possess these adhesive proteins because it is crucial for mussels to adhere to almost any surface in an aqueous environment. This application of biomimicry is a highly promising antibacterial biomaterial coating and tissue adhesion.^35^

Although Ag NP‐based hydrogels have so many advantages, their applications are still far from what is expected. They are less effective in G+ bacteria compared to G− bacteria due to the high resistance from the peptidoglycan within the cell walls of G+ bacteria.^36^ Furthermore, the development of nanoparticles was largely restricted because of their physical and chemical instability, therefore stabilization of metallic nanosystems will become a promising area of research within nanoscience and nanotechnology.

Although Ag ions are efficient bactericides at a concentration of as low as ≈0.001–0.05 ppm, their tissue toxicity and cytotoxicity should be discussed. The serum albumin in human blood can reduce the antibacterial effect of Ag NP‐embedded hydrogels as well.^37^ In addition, it is reported that Ag NPs resulted in several negative impacts on genes. The balance between anti‐reactive oxygen species (ROS) response and DNA damage and the balance between mitosis inhibition and chromosome instability may play significant roles in Ag‐induced toxicity.^38^ Thus, there is a preference to minimize the toxicity and reduce the influence of serum albumin when designing Ag NP‐based hydrogels. Additionally, more nontoxic and environmental‐friendly synthesis strategies of Ag NP‐based hydrogels, such as the size‐controllable synthesis of Ag NPs with tobacco mosaic virus as a biomediator without any external reducing agents should be developed.^39^

#### 2.1.2 Gold Nanoparticle‐Loaded Hydrogels

Although Au is universally considered to be biologically inert, gold nanoparticles (Au NPs) have diverse biological functions. Au NPs play a significant role in biological applications, such as cell imaging, photothermal therapy, sensing, and antimicrobial activities.^40^ Au NPs can be designed to be various sizes and functionalized with desired polymers, thus recognized as biocompatible materials. Daniel‐da‐Silva et al. developed Au/gelatin hydrogel nanocomposites, which were crosslinked with genipin. When triggered by thermal stimuli, the composites had the potential for release of the encapsulated Au NPs.^41^ Au NPs possess antibacterial capability by attaching to bacterial membranes, thus leading to the leakage of bacterial contents or the penetration of the outer membrane and peptidoglycan layers, resulting in bacterial death. Au NPs also reverse bacterial resistance to some extent when combined with non‐antibiotic or antibiotic molecules.^42^

**Figure 4:**
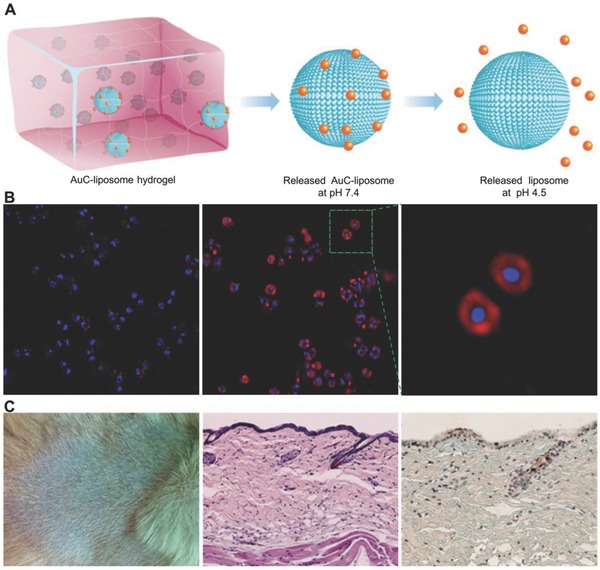
Hydrogel containing nanoparticle‐stabilized liposomes for topical antimicrobial delivery. A) Schematic illustration of hydrogel containing nanoparticle‐stabilized liposomes for topical antimicrobial delivery. B) Fluorescence study of fusion interaction between AuC–liposome hydrogel and *S. aureus* bacteria. C) The toxicity evaluation of AuC–liposome hydrogel using a mouse skin model. Reproduced with permission.^45^ Copyright 2014, American Chemical Society.

However, compared with Ag NP‐loaded hydrogels, the antibacterial Au NP‐loaded hydrogels remain insufficiently explored. The *N*‐isopropylacrylamide‐based hydrogels containing Au NPs^43^ and the pH‐responsive poly(methacrylic acid) hydrogel microcapsules as Au NP nanoreactors^44^ have been reported, but their antibacterial properties remain unstudied. Gao et al. demonstrated that hydrogels containing Au NP‐stabilized liposomes displayed excellent antibacterial properties on *S. aureus* without skin toxicity to mice (**Figure** **4**).^45^ In their research, the carboxyl‐modified Au NPs were absorbed onto the outer surfaces of cationic liposomes as stabilizers. The hydrogel formulation allowed for controllable viscoelasticity and tunable liposome release rate. The released Au NPs subsequently fuse with bacterial membranes in a pH‐dependent manner. In summary, the hydrogel formulation exhibited great promise for applications against various microbial infections. Furthermore, in order to obtain better antibacterial properties, some researchers fabricated bimetallic (i.e., Ag and Au) hydrogel nanocomposites, which achieved the desired antibacterial activity. Varaprasad et al. prepared the dual‐metallic (Ag^0^–Au^0^) nanoparticle‐loaded hydrogels through a green process with mint leaf extracts as the hydrogel networks, which exhibited significant antibacterial activity against *Bacillus* and *E. coli*.^46^

#### 2.1.3 Other Metal Nanoparticle‐Loaded Hydrogels

**Figure 5:**
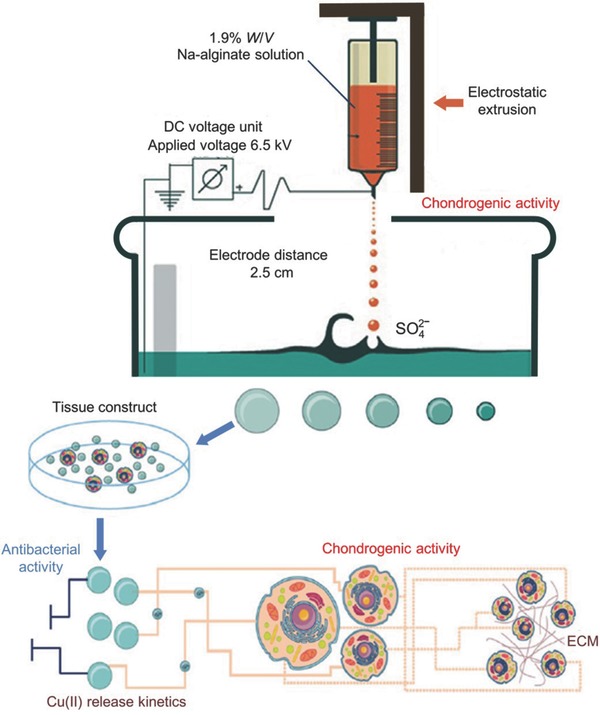
Study and potential biomedical application of Cu–SA. Reproduced with permission.^49^ Copyright 2016, IOP.

Apart from these commonly used metal nanoparticles, the antibacterial cobalt‐exchanged natural zeolite (ZEO)/PVA hydrogels were proved to possess antibacterial activity against *E. coli*.^47^, ^48^ ZEO/PVA hydrogel with 0.48 wt% and higher cobalt‐exchanged ZEO contents showed efficient antibacterial activities against G− bacteria (i.e., *E. coli* and *S. aureus*). Cu–SA hydrogels prepared through electrostatic extrusion were bactericidally effective against *E. coli* and MRSA (**Figure** **5**).^49^ Cu–SA hydrogels, with higher Cu(II) loading (≈100 × 10^−6^ m), were produced by electrostatic extrusion using gelling solutions with Cu(II). The Cu–SA hydrogels exhibited immediate bactericidal effects against *S. aureus* and *E. coli*.

Generally, metallic nanoparticles can attach to and destroy the integrity of bacterial membranes, leading to the leakage of bacterial contents, such as nucleic acids, through the outer membrane and peptidoglycan layer, resulting in the inhibition of protein synthesis. However, the mechanisms behind the antibacterial effects of metallic nanoparticles have not been confirmed. Despite being used in low concentrations, the toxicities of the metal‐based materials remain a major concern. Further studies are required to investigate the effects of particle size, morphologies, surface properties, associated signal transduction mechanisms, and applied concentration of metallic nanoparticles on antibacterial properties. Many of the aforementioned metals, including their alloys and the metal nanoparticles that are applied in modern medical biomaterials, need to be further explored. However, despite the previously discussed questions and challenges, hydrogels provide a convenient and controllable platform for the production of biocompatible functionalized metal nanoparticles.

### 2.2 Metallic Oxide Nanoparticle‐Loaded Hydrogels

In addition to the metal nanoparticle‐loaded hydrogels, metallic oxide nanoparticle‐loaded hydrogels also possess good antibacterial properties. The antibacterial mechanism of metallic oxide nanoparticles differs from metal nanoparticles. The photocatalysis is the main antibacterial mechanism of metallic oxide nanoparticles.^50^ Under the ultraviolet irradiation of sunlight, large amounts of free radicals, namely hydroxyl radicals and oxygen radicals, are produced at the surface of metallic oxide nanoparticles. When the free radicals are exposed to microorganisms, the organic matter of the microorganisms are oxidized into carbon dioxide, therefore the metallic oxide nanoparticles can kill microorganisms in a relatively short amount of time.

Among the various metallic oxides, ZnO is the most popular antibacterial agent.^51^, ^52^, ^53^ ZnO NPs are widely used in many cosmetic materials because they exhibit antibacterial activity and non‐cytotoxicity at the appropriate concentrations. Sudheesh Kumar et al. developed CT hydrogel/ZnO composite bandages for wound healing and collagen deposition.^54^ They are effective against both G+ and G− bacteria as well as high‐temperature resistant and high‐pressure resistant bacterial spores.^55^ Similar to Ag NP‐loaded hydrogels, a composite bandage of SA hydrogel loading ZnO NPs prepared by Mohandas et al. exhibited enhanced swelling, blood clotting, and antibacterial activity. The hydrogel/ZnO NP composite bandage exhibited excellent antimicrobial activities against various strains of bacteria (e.g., *E. coli*, *S. aureus*, *Candida albicans*, and methicillin‐resistant *S. aureus*). When utilized on human dermal fibroblast cells, the composite bandage was nontoxic at low concentrations of ZnO.^56^

The antibacterial hydrogel coatings made from ZnO NP‐incorporated poly(*N*‐isopropylacrylamide) (PNIPAM) were demonstrated to be effective alternatives for biomedical device coatings. This composition of hydrogel exhibited bactericidal activity against *E. coli*.^57^ Furthermore, the hydrogel coatings showed no cytotoxicity toward the mammalian cell line (3T3) over one week. Yadollahi et al. fabricated CMC/CuO (CMC, carboxymethyl cellulose) nanocomposite hydrogels via in situ formation of CuO NPs within swollen CMC hydrogels. The resultant hydrogels exhibited excellent antibacterial effects against both G+ and G− bacteria.^58^ Archana et al. reported that the TiO_2_ NP‐loaded CS–pectin composite hydrogel generated wound dressings with photoactive property, excellent biocompatibility, good antibacterial ability, and enhanced wound closure rate.^59^

Inorganic antibacterial agent‐loaded hydrogels have relatively stable antibacterial properties and high temperature resistance. Unfortunately, their biocompatibilities are unsatisfactory for human implantation. As an alternate, the organic antibacterial agents are used in synthetic antibacterial hydrogel. Organic antibacterial agent is generally classified into small molecule antibacterial agents and polymer antibacterial agents. The hydrogel matrix can be composed of natural polymers and their derivatives, in particular ST, gelatin, CS, CMC, SA, as well as synthetic polymers including PVA and PVP.

## 3 Antibiotic‐Loaded Hydrogels

Although discovered after antibacterial metal agents, antibiotics are undoubtedly the most common and effective antibacterial agents.^60^ However, the drug‐resistant effect that bacteria possess has been the biggest obstacle in the development and applications of antibiotics. To overcome this, it is more promising and practical to minimize the dosage of conventional antibiotics rather than to explore new antibiotics.^61^ Local antibiotic administration, by delivering the adequate bactericidal dose of antibiotics directly into the infected site without significantly overtaking the systemic toxicity level, has drawn increasing attention in recent years.^62^ In biomedical research, fibers, beads, gels, and many other materials are used to deliver antibiotics. Hydrogels, a form of local administration matrix, offer a high surface area to volume ratio and structural controllability, such as porosity to mimic natural tissues. As a result, it is easy for hydrogels to selectively release their loaded drugs at desirable sites,^63^, ^64^ while maintaining high water content and biocompatibility.^65^ Some of the antibiotic‐loaded hydrogels are summarized in the following sections.

### 3.1 Ciprofloxacin‐Loaded Hydrogels

Ciprofloxacin (CIP) is a fluoroquinolone‐based antibacterial agent, with a broad antibacterial spectrum against both G+ and G− bacteria. The partition coefficient (log*P*, octanol–Tris model) of CIP is −1.31^5^.^66^ This is the gold standard for various topical applications, such as for eye and skin infections.^66^, ^67^ The antibacterial mechanism of CIP relies upon the blockage of bacterial DNA duplication by binding to the DNA gyrases and causing double‐stranded ruptures in bacterial chromosomes. Thus, the drug resistance to this antibiotic develops slowly.^68^ The toxicity of CIP is dosage‐related and excessive doses can cause damage to tissues. Utilizing hydrogels as a local delivery system can sufficiently resolve this issue.

CIP can be self‐assembled with a tripeptide (d‐Leu‐Phe‐Phe) and incorporated into antibacterial nanostructured hydrogels with high drug loading efficiency (DLE) and a prolonged release.^64^ This CIP–peptide self‐assembled hydrogel showed high antimicrobial activity against *S. aureus*, *E. coli*, and *Klebsiella pneumoniae*. Furthermore, no cytotoxicity was found in hemolysis assays of red blood cells or in cultures of fibroblast cells. Two electrosynthesized polyacrylate hydrogels loaded with CIP prevented the Ti implant‐associated infections. The antibiotic‐modified hydrogel coatings had a long‐term release property, which exhibited antimicrobial activity against MRSA and good biocompatibility with MG63 human osteoblast‐like cells.^62^ A hydrogel generated by the polymerization of 3‐aminophenylboronic acid with PVA for CIP loading was reported to facilitate wound healing in diabetes patients.^69^ The hydrogel composite exhibited an ability to bind glucose and release CIP, which demonstrates the possibility of using it for wounds, particularly in diabetic patients. Colon‐associated diseases like constipation were reported to be treated with hydrogels containing laxative psyllium and CIP. The hydrogel with laxative action of psyllium and slow release of CIP exhibited a satisfactory therapeutic effect for treatment of diverticulitis.^70^ A liposomal hydrogel containing CIP was reported to improve the maximum ocular availability in the cornea of albino rabbits.^71^ Shi et al. conjugated CIP to the hydrogel network structure and obtained a composite hydrogel with ultraviolet‐triggered CIP release behavior. The composite hydrogel showed excellent antibacterial effects against MRSA.^72^

### 3.2 Gentamicin‐Loaded Hydrogels

**Figure 6:**
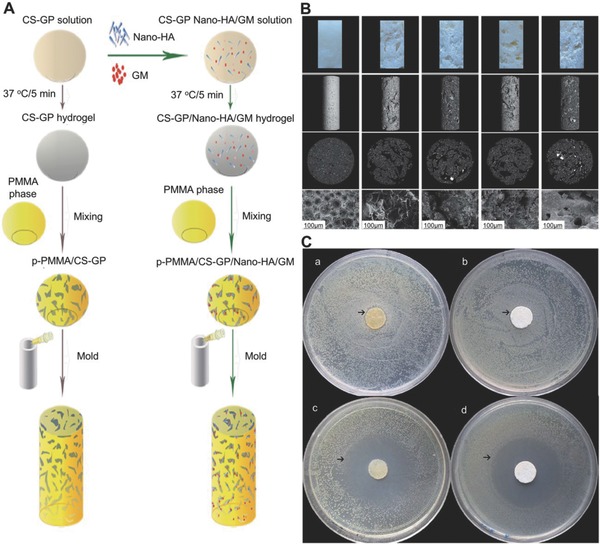
Beneficial effects of biomimetic nHA/GEN‐enriched CS–GP hydrogel on performance of injectable PMMA. A) Synthetic process of PMMA‐based cements. B) Morphology of the GS–GP hydrogel. C) Antibacterial activity of samples by zone of inhibition test. Reproduced with permission.^74^ Copyright 2015, Royal Society of Chemistry.

Gentamicin (GEN) is a traditional broad‐spectrum antibiotic used for the treatment of infections of the skin, soft tissues, and wounds, but its systemic toxicity (e.g., kidney) and low plasma concentration remain a problem, which hinders its applications. Local administration of functional GEN hydrogels offers an efficient solution. Posadowska et al. fabricated an injectable drug delivery system, which consists of GEN‐loaded poly(lactide‐*co*‐glycolide) (PLGA) NPs embedded in the gellan gum hydrogel. The system was suitable for injection and was antibacterially active against *Staphylococcus saprophyticus* without affecting the bone forming cells.^73^ Sa et al. developed a class of thermosensitive CS–GP hydrogels incorporating nanosized hydroxyapatite (nHA)/antibiotic GEN. The thermosensitive hydrogels were introduced into polymethylmethacrylate (PMMA) bone cement, resulting in an increased mineralization capacity and an enhanced antibacterial activity of the cement (**Figure** **6**).^74^

GEN‐loaded PVA and PVA–AAm hydrogels crosslinked by *Sterculia* can be a form of potent antibacterial wound dressings due to their good biomedical properties, specifically blood compatibility, tensile strength, burst strength, water vapor permeability, and oxygen diffusion.^75^, ^76^ Superabsorbent polysaccharide GEN hydrogels based on pullulan derivatives also present a broadened view about antibacterial hydrogels. The ability to expand to 4000% of its initial volume provides the hydrogels with a quick hemostatic ability and a capacity to prevent the wound bed from accumulation of exudates.^77^ Phospholipid‐modified solid lipid microparticles encapsulating GEN were loaded into different polymer hydrogels. Among them, poloxamer 407 microgels displayed the most desirable properties specifically rapid antibacterial activity, in vitro diffusion‐dependent permeation, ability to spread, and appropriate viscosity.^78^ These results indicated that the same drug can achieve different diffusion speeds on hydrogels due to the different matrices being employed.

### 3.3 Vancomycin‐Loaded Hydrogels

**Figure 7:**
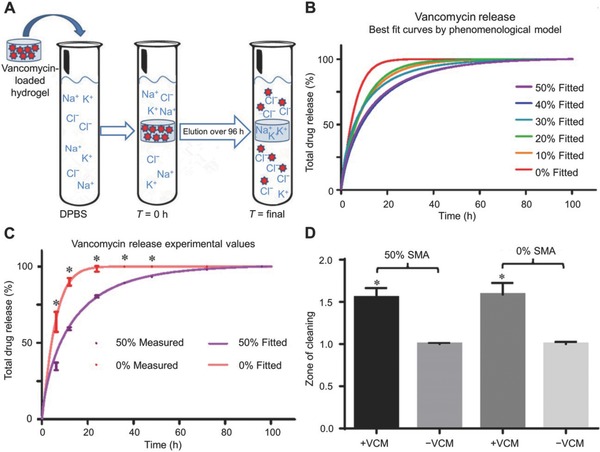
Controlled delivery of VAN via charged hydrogels. A) VAN release from charged hydrogels. B) Best fit of data as calculated by phenomenological mathematical model described in text. C) Comparison of fitted model to obtained data points for VAN‐loaded and ‐unloaded (0% and 50% sodium methacrylate (SMA)) hydrogels. D) Zone of clearing assay comparing 0% and 50% SMA. Reproduced from ref. 80.

Clinically, vancomycin (VAN) is an antibiotic that is considered as the last form of defense against an infection. However, VAN‐resistant *Enterococcus* was recently discovered.^79^ As mentioned above, utilizing hydrogels as a drug delivery system protects and enhances the effectiveness of VAN. Gustafson et al. developed a charged hydrogel as a carrier. The charged hydrogel, which was loaded with VAN over 500 µg mg^−1^ hydrogel, was able to control the VAN delivery and was used to combat the surgical site infections against MRSA (**Figure** **7**).^80^ Development of an injectable gellan gum‐based PLGA NP‐loaded system,^81^ injectable Pluronic–α‐CD supramolecular gels (CD, cyclodextrin),^82^ and hydrogels consisting of thiolated chitosan crosslinked with maleic acid‐grafted dextran^83^ provided new opportunities for antimicrobial research. The photo‐crosslinked methacrylated dextran and poly(l‐glutamic acid)‐*graft*‐hydroxyethyl methacrylate (PGA‐*g*‐HEMA) hydrogels were studied^84^ and both exhibited excellent antibacterial properties and desirable release capabilities.

### 3.4 Other Antibiotic‐Loaded Hydrogels

In addition to the aforementioned antibiotic‐loaded hydrogels, other antibiotic‐loaded hydrogels were developed as well. Ampicillin sodium‐loaded PVA–SA hydrogel exhibited strong antibacterial property to both G+ and G− bacteria and improved hemolysis.^85^ Cephalosporin is a widely used neutrapen‐resistant and broad‐spectrum β‐lactamase‐based antibiotic.^86^ Hydrogels containing cefditoren pivoxil achieved gastroretentive effect^87^ and methoxy poly(ethylene glycol)‐*co*‐poly(lactic acid‐*co*‐aromatic anhydride) hydrogels containing cefazolin offered a stable release profile without an initial burst release and effective antibacterial properties against *E. coli*.^88^ Levofloxacin‐loaded hyaluronic acid hydrogels were reported to be able to attack bacteria within the cells for both *S. aureus* and *P. aeruginosa* strains.^61^ A hydrogel based on (−)‐menthol, which is a traditional cooling compound followed by an amino acid derivative through an alkyl chain, provided an innocuous environment to living cells and was able to deliver lincomycin to the local infection site.^89^ Furthermore, the hydrogel composites were completely innoxious to HeLa cells. Doxycycline (DOX) was also loaded in situ into a thermosensitive hydroxypropyl‐β‐cyclodextrin (HP‐β‐CD) hydrogel for ophthalmic delivery.^90^ The release of DOX from hydrogel followed a zero order equation, suggesting that it occurred due to corrosion of the poloxamer hydrogel. The liposomes‐in‐hydrogel delivery systems can control and prolong the release of mupirocin (MIP). MIP is a promising antibiotic that is well tolerated in topical administration with minimized side effects and leads to improved burn therapy.^91^

## 4 Biological Extract‐Loaded Hydrogels

Biological extracts include extracts from plants and animals.^92^ Seaweed extract‐based hydrogel was reported as an antibacterial wound dressing.^93^ PVA composite hydrogels based on combinations of agar and carrageenan have been proved to be useful as wound dressings in the treatments of burns, nonhealing ulcers of diabetes, and other external wounds.^94^ Although some studies have stated that SA does not display antibacterial properties, it can be an ideal material for wound dressings due to its morphology, fiber size, porosity, degradation, and swelling ratio.^93^, ^95^ Allicin–CS complexes were proved to be active against bacteria involved in spoilage and can be used as an antibacterial agent in foods.^96^ Curcumin (CUR), a nontoxic and bioactive agent found in turmeric, has been applied for centuries as a remedy to various ailments.^97^ However, its applications were limited by its low aqueous solubility and poor bioavailability. As a result, hydrogels incorporated by CUR nanoparticles were developed. Ag NP–CUR loaded hydrogels utilized for wound dressings were reported to exhibit good antibacterial property and sustained release, which indicated enormous therapeutic values.^98^, ^99^ SA hydrogels encapsulated with essential oils, such as lavender, thyme oil, peppermint, tea tree, rosemary, cinnamon eucalyptus, and lemongrass, were reported to be qualified as disposable wound dressings due to the distinctive antibacterial properties of essential oils.^100^

Among the biological extracts from animals, honey was the most easily acquired. Honey showed an antimicrobial activity in the management of various wounds.^101^ CMC hydrogels incorporated with propolis honey were prepared by gamma radiation to produce a functional wound dressing.^102^ Hydrogel contact lenses incorporated with lysozymes derived from normal tears exhibited remarkable antibacterial activity due to the inherent antibacterial property of lysozymes.^103^ Vitamin E (VitE) is an important antioxidant and biodegradable extract. Hydrogels of VitE‐functionalized polycarbonates for antibacterial applications displayed an excellent compatibility with human dermal fibroblast. It can be loaded with cationic polymers and/or fluconazole at minimum biocidal concentrations to kill bacteria and fungi.^104^

Polysaccharides with antibacterial ability are often natural macromolecules or their derivatives, such as ST and CS, which are frequently used for the preparation of hydrogels because of their nontoxicity, biodegradability, biocompatibility, and abundance in nature.^105^, ^106^ Some of these polysaccharides have inherent antibacterial activity. Among them, CS is the most popular polysaccharide. CS has a wide antibacterial spectrum and high killing rate against G+ and G− bacteria while displaying a low toxicity toward mammalian cells.^107^ CS can dissolve in weakly acidic solution and release NH_2_^+^, then bind with negatively charged macromolecules on the microbial cell surface to achieve bacterial stasis.^108^ The polymers mainly composed of CS and semi‐interpenetrating carboxymethyl chitosan (CMCS)/polyacrylonitrile hydrogels were reported to present good antibacterial activity when the CMCS content was increased.^109^ Hydrogel coatings prepared by electrophoretic codeposition of CS/alkynyl CS exhibited better antibacterial activities than pure CS hydrogel.^110^ CS‐grafted polymer‐based hydrogels containing mica nanocomposite produced a rougher surface while maintaining antibacterial activity.^111^ These biological extracts are easy to obtain, handle, possess excellent biocompatibilities, and good antibacterial properties, making them promising antibacterial biomaterials.

## 5 Synthetic Antibacterial Drug‐Loaded Hydrogels

Synthetic antibacterial drugs discussed here refer to the nitroimidazoles, sulfanilamide groups, and other frequently used drugs, but do not include semisynthetic antibiotics nor biological extracts. Although the special chemical structures benefit synthetic drugs significantly, they carry risks and damages to the normal tissues as well. A stable and safe delivery system for them is necessary.

Hydrogel composed of dextrin and PAA was utilized for the delivery of ornidazole, which is a nitroimidazole‐derived antibacterial drug used for the digestive system. It showed effects on anaerobic bacteria and amoeba^112^ with pH‐ and temperature‐controllable release profiles.^113^ Moreover, the hydrogel with degradable characteristics showed no cytotoxic behavior toward human mesenchymal stem cells. Hydrogels based on dextrin grafted with poly(2‐hydroxyethyl methacrylate) (PHEMA) were also good candidates for the orally administered drug delivery system in the colon region.^65^ CS/gelatin/β‐GP hydrogel containing metronidazole was tested as an injectable form for periodontal infection. It was able to maintain the release of metronidazole in concentrations that were effective for killing pathogenic bacteria of *Clostridium sporogenes*.^114^ PAA–CS composite hydrogels containing tinidazole (TIN) and theophylline also have been studied to control and sustain TIN and theophylline delivery.^115^ Simply put, in the presence of CS, the acrylic acid and *N′*‐methylene bis‐acrylamide were crosslinked by radical copolymerization to synthesize the composite hydrogels. CHX is considered to be a promising antibacterial agent that possesses a broad antibacterial spectrum including both G+ and G− bacteria.^116^ CHX‐contained poly(ethylene glycol)‐*block*‐poly(l‐lactide) nanoparticles were loaded in hydroxyethyl cellulose hydrogel, allowing the hydrogel system to enhance antibacterial activity against *Enterococcus faecalis* for root canal system disinfection.^117^ CS‐HTCC/GP‐0.1% CHX (CS, quaternized CS, and α,β‐GP loading with 0.1% CHX (w/v)) thermoresponsive hydrogels showed an excellent antibacterial effect against *Porphyromonas gingivalis*, *Prevotella intermedia*, and *Actinobacillus actinomycetemcomitans*.^118^ Chlorhexidine diacetate‐contained poly(2‐hydroxyhexyl methacrylate‐*co‐N‐*isopropylacrylamide) hydrogels are a promising thermoresponsive and antibacterial biomaterial (e.g., *Staphylococcus epidermidis* (*S. epidermidis*)).^119^ Wound dressings composed of OCT‐loaded nanocellulose were proven to possess antibacterial activity with minimized side effects.^120^ This OCT‐loaded nanocellulose exhibited a slower OCT release rate of up to 96 h, which demonstrated high biocompatibility in human HaCaT keratinocytes and antimicrobial activity against *S. aureus*. PHEMA‐conjugated β‐CD or directly crosslinked HP‐β‐CD hydrogels were applied to load TSC, an antibacterial drug used in ophthalmic diseases for fabricating antibacterial soft contact lenses.^121^ Cetylpyridinium chloride‐immobilized PVA hydrogel offered a sustained release profile for wound therapy.^122^ Chloramine‐T and sulfadiazine sodium coloaded hydrogels composed of PVA, PVP, and glycerin showed an excellent swelling capacity, which accelerated the wound healing with an antibacterial effect.^123^ A PVP–iodine hydrogel was found to enhance the epithelialization and reduce the loss of skin grafts in wound therapy.^124^ Poly(*N*‐hydroxyethyl acrylamide) (PHEAAm)/SAL hydrogels provided both antibacterial and antifouling functions.^125^ This research showed that SA‐treated PHEAAm hydrogels could inhibit both G+*E. coli* RP437 and G−*Staphylococcus epidermidis*. Alginate hydrogel spheres releasing ITZ achieved long‐term antibacterial activity by improving the alkali and heat resistance abilities.^126^ Evidence indicates that the synthetic drug‐loaded hydrogels could achieve desirable drug delivery as well as avoid risks and minimize side effects. It was of equal importance that hydrogels offer potential for widespread application of antimicrobial and antiviral agents.

## 6 Carbon Material‐Loaded Hydrogels

Some carbon materials combined with hydrogels were developed for inhibition of bacteria. Venkatesan et al. prepared CT–carbon nanotube hydrogels by freeze‐lyophilization method, which exhibited antimicrobial activity against *S. aureus*, *E. coli*, and *Candidatropicalis*.^127^ Composite CT/active carbon hydrogels prepared by ammonia vapor treatment showed an potential application to be used as wound dressings.^128^ Graphene oxide (GO) also has immense potential in the antibacterial field. A facile one‐pot method was used to synthesize GO‐based hydrogels (i.e., benzalkonium bromide/GO hydrogel and benzalkonium bromide/polydopamine/reduced GO hydrogel), which exhibited strong antibacterial activity against G+ and G− bacteria.^129^ Zeng et al. prepared an Ag/reduced GO hydrogel by a facile hydrothermal reaction, which consisted of two parts: (i) a controlled porous reduced GO network and (ii) well‐dispersed Ag NPs.^130^ The antibacterial hydrogels were generated by crosslinking the Ag/graphene composites with acrylic acid and *N,N′*‐methylene bisacrylamide, which exhibited good antibacterial abilities against *E. coli* and *S. aureus*. The excellent biocompatibility, high swelling ratio, and good extensibility were also found in this hydrogel system.^131^

## 7 Hydrogels with Inherent Antibacterial Activity

Hydrogels with inherent antibacterial activity discussed here refer to the hydrogels that contain antibacterial components.^132^ These hydrogels, with inherent antibacterial activity, were developed in recent years as effective antibacterial agents with little or even no side effects compared to the traditional ones. The main forms of these hydrogels are discussed below.

### 7.1 Hydrogels with Antibacterial Polymers

Antibacterial polymers include nonstimulated antibacterial polymers and potential antibacterial polymers. The most common nonstimulated antibacterial polymers have certain components in their structures that are important for antibacterium. The hydrogels composed of thermoresponsive PNIPAM and redox‐responsive polyferrocenylsilane macromolecules exhibited strong antibacterial activities while maintaining high biocompatibilities.^133^ The redox‐induced formation of hydrogel–Ag composites showed a good antimicrobial activity against *E. coli*. pH‐sensitive and thermal‐sensitive hydrogels based on HEMA and IA copolymers possess potential biomedical applications, especially for skin treatments and wound dressings.^134^ P(HEMA/IA) could block the entry of *S. aureus* and *E. coli* into hydrogel dressing. In addition, no evidence of cell toxicity or considerable hemolytic activity was observed in an in vitro study of P(HEMA/IA) biocompatibility. Hydrogels prepared by the photopolymerization of PEG diacrylate and a monomer containing ammonium salt (RNH_3_Cl) demonstrated both antibacterial and antifouling properties.^135^ The potential antibacterial polymers are a class of polymers that could be converted to become antibacterial under certain conditions, such as exposure to light. The photodynamic poly(2‐hydroxyethyl methacrylate‐*co*‐methyl methacrylate) (P(HEMA‐*co*‐MAA)) copolymers crosslinked by porphyrin were reported to be promising for the prevention of intraocular lens‐associated infectious endophthalmitis.^136^ Another photodynamic PHEMA‐based hydrogel also exhibited light‐induced bactericidal effect through the release of nitric oxide.^137^ These antibacterial polymers provided not only antibacterial materials but also responsive delivery and release methods.

### 7.2 Hydrogels with Antibacterial Peptides

Antibacterial peptides (AMPs) are an abundant and diverse group of molecules produced by many types of tissues and cells in plant and animal species.^4^ They are recognized as a possible source of panacea for the treatment of antibiotic‐resistant bacterial infections.^138^, ^139^ AMPs have strong antibacterial activities against a very broad spectrum of microorganisms, including G+ and G− bacteria, fungi, and even viruses.^140^

**Figure 8:**
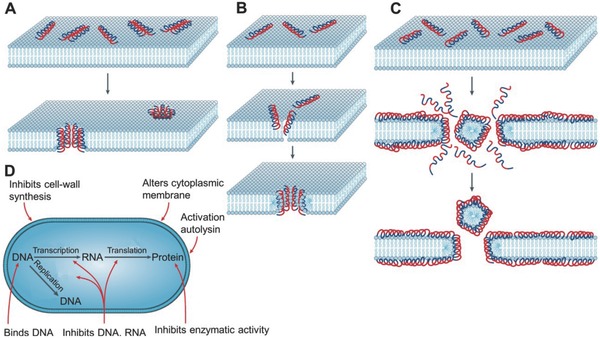
Antimicrobial peptides: pore formers or metabolic inhibitors in bacteria. A) Barrel‐stave model, B) toroidal model, and C) carpet model of antimicrobial peptide‐induced killing. D) Mode of action for intracellular antimicrobial peptide activity. Reproduced with permission.^138^ Copyright 2005, Nature.

It is generally accepted that the antibacterial mechanism of AMPs is that they associate with the membrane leading to disruption of the bacterium (**Figure** **8**).^138^ Bai et al. designed an amphiphilic peptide A_9_K_2_ that could effectively inhibit both G+ and G− bacterial strains.^141^ The enzymatic A_9_K_2_ hydrogel possessed good biocompatibility and showed excellent selectivity by favoring the adherence and spreading of mammalian cells. Baral et al. prepared an antibacterial dipeptide, which showed excellent antibacterial activity against G− bacteria (*E. coli* and *P. aeruginosa*), as well as high biocompatibility with human red blood cells and human fibroblast cells.^142^ Wang et al. fabricated enzymatically crosslinked ε‐poly‐l‐lysine hydrogels, which exhibited efficient antibacterial activity against both G+ and G− bacteria.^143^ Peptide‐based β‐hairpin hydrogels were reported with MAX1 peptides by Salick et al. in 2007^144^ and with arginine‐rich peptides by Veiga et al. in 2012.^145^ Both of them are self‐assembly peptides that exhibited potent antibacterial activity.

The self‐assembled peptides comprised of two antibacterial peptides (KIGAKI)_3_—NH_2_ and a central tetrapeptide linker can maintain a stable β‐hairpin structure.^146^ Polylysine, a popular AMP reported by Zhou et al., has been applied in photopolymerized antibacterial hydrogels, which generated promising coatings for medical devices and implants.^147^ In addition to antibacterial peptide maximin‐4‐loaded PHEMA hydrogels,^148^ l‐cysteine‐ and silver nitrate (AgNO_3_)‐loaded hydrogels were proved to possess antibacterial activity against *Staphylococci*, *Bacilli*, *Escherichia*, and *P. aeruginosa* strains.^149^

**Figure 9:**
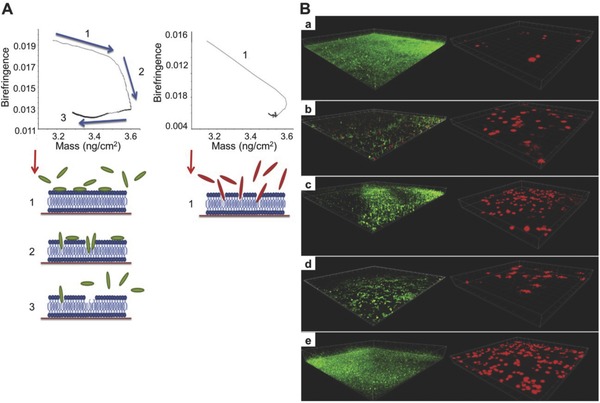
Comparative surface antimicrobial properties of synthetic biocides and human apolipoprotein E‐derived antimicrobial peptides. A) Dual‐polarization interferometer associated with mass uptake of ApoEdpL‐W (a peptide derivative of human apolipoprotein E) and polyhexamethylene biguanide (PHMB). B) CLSM images showing the growth of *P. aeruginosa* and L929 cell line exposed to a PHEMA hydrogel that has been previously exposed to (a) phosphate‐buffered saline (PBS), (b) ApoEdpL‐W, (c) CHX, (d) PHMB, and (e) triclosan. Reproduced with permission.^151^ Copyright 2013, Elsevier.

Although the hydrogels with AMPs displayed some disadvantages, such as tissue toxicity and hemolysis,^150^ they are still attractive due to their increased antibacterial ability and biocompatibility when compared to synthetic drugs with similar structures (**Figure** **9**).^151^ Extensive research is being carried out to improve the biocompatibility. Specifically, a hydrogel of cell adhesive polypeptides and PEG with inherent antibacterial activity was developed by Song et al. as a potential scaffold for cutaneous wound healing.^152^ The hydrogel formed by crosslinking poly(Lys‐Ala) with 6‐arm poly(ethylene glycol)‐amide succinimidyl gluta exhibited significant antibacterial activity against *S. aureus* and *E. coli*. Moreover, a protein anchor developed to immobilize functional protein to poly(ethylene glycol) diacrylate microspheres in 2013 proved to be a fascinating method to maintain therapeutic efficacy without toxicity.^153^

### 7.3 Amphoteric Ion Hydrogels

Amphoteric ion hydrogel works similarly with AMPs. The electrostatic interactions facilitate the bindings between the polymers and anionic bacterial membranes, resulting in the physical destruction of membrane structures and cell death.^154^

QA compound is one of the most famous antibacterial materials. Antibacterial hydrogels containing QA groups synthesized by Zhou et al. through a facile thiol–ene “click” reaction exhibited excellent antibacterial efficacy against MRSA.^155^ A cellulose (CEL)‐based hydrogel containing QA groups were prepared by Peng et al. via a simple chemical crosslinking and showed strong antibacterial ability against *Saccharomyces cerevisiae*.^156^ He and co‐workers designed photo‐crosslinked polymer ionic hydrogel films incorporating QA chloride groups, which exhibited antibacterial ability against *E. coli* with almost 100% killing efficiency.^157^

PEG hydrogel networks incorporated by polycarbonate via Michael addition by Liu et al. were reported to have more than 99.99% efficiency against MRSA.^158^ It is worth mentioning that antimicrobial and nonfouling hydrogel did possess significant skin toxicity or hemolytic activity. When combined with hydrogels, the amphiphiles perform the same as the AMPs. Polyampholytic hydrogels with high antibacterial activity can exhibit high water absorbency.^157^ Potent antibacterial hydrogels based on anti‐inflammatory *N*‐fluorenyl‐9‐methoxycarbonyl amino acid/peptide‐functionalized cationic amphiphiles exhibited efficient antibacterial activity against both G+ and G− bacteria.^159^

To achieve a bifunctional hydrogel with both antibacterial and antifouling capacities, a zwitterionic hydrogel was conjugated with an antibacterial agent, SAL. The resultant hydrogel can reach one‐SAL‐per‐monomer DLE while maintaining the nonfouling property at protein and bacteria levels.^160^ To improve the biocompatibilities of amphiphiles, Dutta et al. developed cholesterol‐based amino acid containing hydrogels. Ag NPs were synthesized in situ and the amphiphile–Ag NPs soft nanocomposite exhibited notable antibacterial property.^161^ In addition to the hydrogels developed against normal G+ and G− bacteria, an anti‐mycobacterial supramolecular hydrogel based on amphiphiles was developed by Bernet et al. It retains specific and chain‐length dependent antibacterial and anti‐mycobacterial activity while showing no antiproliferative and negligible cytotoxic effects.^162^

## 8 Hydrogels with Synergetic Effects

Hydrogels with synergetic effects refers to hydrogels containing two or more antibacterial agents, which can enhance antibacterial effects. Metal nanoparticles and antibiotics are commonly reported to be incorporated into hydrogels together to obtain synergetic effects. In addition, as described above, the combined utilization of Ag NPs and reduced GO is also a common mechanism to enhance antibacterial effects.^130^, ^131^

### 8.1 Synergetic Effective Hydrogels Containing Metal Nanoparticles

Metal nanoparticles in synergetic effective hydrogels were mainly Ag NPs. Ag NPs can be loaded into synthetic amphiphiles, amino acids, and even biological extract‐based hydrogels.^163^ Ag NP composite systems are more suitable for biomedical applications because of their good biocompatibility with biological molecules, cells, and tissues.^164^

Amphiphilic hydrogels with in situ‐synthesized Ag NPs reported by Dutta et al. exhibited improved biocompatibility and antibacterial efficacy, which are promising in applications of biomedicine and tissue engineering.^165^ They also reported self‐assembly amino acid‐based amphiphilic hydrogels containing in situ‐synthesized Ag NPs, which exhibit lethal bactericidal activity toward both G+ and G− bacteria while maintaining the growth of mammalian cells, which remained unaffected on the surface.^166^

As reported in 2011, the hydrogels based on l‐cysteine and AgNO_3_ were used to prepare bactericidal fibers and fabrics, and the Ag^+^ glutathione hydrogel exhibited improved cytocompatibility.^167^ Furthermore, the composite hydrogel offered more possibilities in potential biomedical applications like wound dressings for burn victims. For other combinations, the antibacterial efficacy of these hydrogel nanocomposites was largely enhanced by the incorporation of both Ag NPs and CUR. The entrapped Ag NPs and CUR molecules were constantly released, thus the hydrogel nanocomposites could be applied in enormous prolonged severe infection therapies.^99^

### 8.2 Synergetic Effective Hydrogels Containing Antibiotics

**Figure 10:**
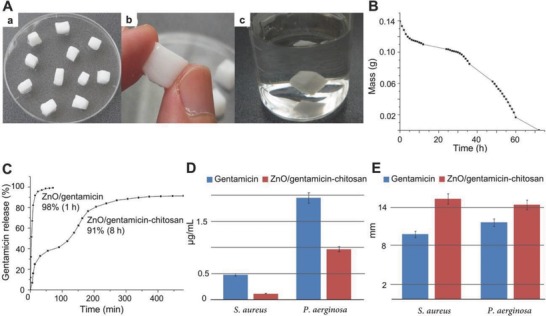
A controlled release ZnO/GEN–CS composite with potential applications in wound treatment. A) (a) Zn–CS gel (12:1) cut in shapes, (b) close view of a ZnO–CS cube, and (c) ZnO–CS cube after three months in water. B) Stability of ZnO–CS gel in laboratory atmosphere. C) GEN release from ZnO/GEN nanopowder and ZnO/GEN–CS gel. D,E) Graphic representation of MICs and inhibition diameters of GEN and ZnO/GEN–CS. Reproduced with permission.^168^ Copyright 2014, Elsevier.

Hydrogels containing antibiotics exhibited more potent antibacterial property and biocompatibility when combined with other antibacterial materials. A ZnO/GEN–CS composite gel with a controlled release profile was reported to be promising in the treatment of wounds. The composite gel of ZnO, GEN, and CS significantly improved the minimum inhibitory concentrations (MICs) against G+ and G− bacteria compared with the GEN control group (**Figure** **10**).^168^ Bacterial CEL polymers grafted by RGDC (R: arginine; G: glycine; D: aspartic acid; C: cysteine) and GEN offered an inspiring and effective antibacterial composite.^169^ CIP was combined with different materials, from metal nanoparticles to amphiphiles, to develop synergetic effective antibacterial hydrogels. Antibacterial nanostructure hydrogels containing self‐assembled CIP and tripeptide were reported by Marchesan et al. and played a significant role in the design of cost‐effective nanomaterials for prolonged drug release.^64^ The magnetically mediated release of CIP‐loaded super‐paramagnetic nanocomposites provided the synergetic effective hydrogels with an effective drug release approach.^68^ The quaternized gellan–CS particles were demonstrated to be potent in sustained release applications of CIP.^170^ In addition, tetracycline hydrochloride/Ag NP composite hydrogels were developed and inhibited bacteria in a simulated colon environment.^171^ All these synergistically effective composite hydrogels offer possible approaches to minimize the dosage of antibiotics required.

## 9 Summary and Prospects

Hydrogels as antibacterial biomaterials can be an alternative and amenable solution to traditional antibiotic treatments. Controlled and prolonged release, local administration, stimulated switch on–off release, enhanced mechanical strength, and improved biocompatibility are all important advantages that a broad diversity of hydrogels can provide and that is exactly what antibacterial biomaterials currently require. Antibacterial hydrogels can be widely applied in the field of wound dressings, urinary tract coatings, catheter‐associated infections, gastrointestinal infections, osteomyelitis, and contact lens. Based on current research regarding the development and application of antibacterial hydrogels, most researchers have been investigating hydrogels composed of polysaccharides, PEG, or other hydrophilic polymers in combination with a variety of bactericidal substances. For hydrogels to be utilized therapeutically, biocompatibility and biodegradability are the utmost important requirements. Furthermore, as a drug carrier, hydrogels should have high DLE. In regards to the side effects, there was no inflammation in the adjacent connective tissue after biodegradation of the hydrogels. Based on the above factors, intelligent hydrogel platforms should be exploited to overcome the challenges of local antibacterial drugs.

Although inorganic antibacterial agents like Ag NPs have good antibacterial properties, the unsatisfactory biocompatibility and dosage dependency limit their applications. Moreover, many drug‐resistant bacteria have evolved because of the misuse of traditional antibiotics and other antibacterial drugs. The special antibacterial mechanism of antibacterial peptides provides a solution to the issue of bacterial resistance. Therefore, fabricating antibacterial peptide hydrogels through the incorporation of antibacterial peptides with hydrogels will be the key to overcome these limitations. Antibacterial hydrogels will finally be able to conquer the vast issues of traditional therapies. Antibacterial biomaterials, their unique combinations, and the approaches currently being developed will provide a promising future for anti‐infection treatment.

## Conflict of Interest

The authors declare no conflict of interest.
